# An Integrated Mechanistic Model of Mindfulness-Oriented Recovery Enhancement for Opioid-Exposed Mother–Infant Dyads

**DOI:** 10.3389/fpsyg.2021.688359

**Published:** 2021-10-28

**Authors:** Sarah E. Reese, Elisabeth Conradt, Michael R. Riquino, Eric L. Garland

**Affiliations:** ^1^School of Social Work, College of Health, University of Montana, Missoula, MT, United States; ^2^Child Adaptation and Neurodevelopment Lab, Department of Psychology, University of Utah, Salt Lake City, UT, United States; ^3^School of Social Welfare, University of Kansas, Lawrence, KS, United States; ^4^Center on Mindfulness and Integrative Health Intervention Development, College of Social Work, University of Utah, Salt Lake City, UT, United States

**Keywords:** pregnancy, parenting (MeSH), opioid misuse, mindfulness, savoring

## Abstract

A growing body of neurobiological and psychological research sheds light on the mechanisms underlying the development and maintenance of opioid use disorder and its relation to parenting behavior. Perinatal opioid use is associated with risks for women and children, including increased risk of child maltreatment. Drawing from extant data, here we provide an integrated mechanistic model of perinatal opioid use, parenting behavior, infant attachment, and child well-being to inform the development and adaptation of behavioral interventions for opioid-exposed mother–infant dyads. The model posits that recurrent perinatal opioid use may lead to increased stress sensitivity and reward dysregulation for some mothers, resulting in decreased perceived salience of infant cues, disengaged parenting behavior, disrupted infant attachment, and decreased child well-being. We conclude with a discussion of Mindfulness-Oriented Recovery Enhancement as a means of addressing mechanisms undergirding perinatal opioid use, parenting, and attachment, presenting evidence on the efficacy and therapeutic mechanisms of mindfulness. As perinatal opioid use increases in the United States, empirically informed models can be used to guide treatment development research and address this growing concern.

## Introduction

In the United States, from 1999 to 2014, the prevalence of maternal opioid use disorder (OUD) at delivery increased four-fold from 1.5 per 1,000 births to 6.5 per 1,000 births (Haight et al., 2018). This dramatic shift has led to adverse consequences for mothers and babies. Pregnant and postpartum women with OUD experience a higher risk of maternal death due to opioid overdose (Haight et al., 2018; Schiff et al., 2018; Smid et al., 2019). For newborns, *in-utero* opioid exposure can result in neonatal opioid withdrawal syndrome, a group of symptoms that occurs when a neonate withdraws from opioids on which they were physiologically dependent (Kocherlakota, 2014). Though the quality of parenting behaviors varies among individuals, opioid use during pregnancy has also been associated with higher risk for neglect and abuse (Smith et al., 2007) and has been cited as a risk factor for child protective services involvement (Leventhal et al., 1997; Burke, 2007; Hafekost et al., 2017; Prindle et al., 2018). Despite these risks, pregnancy is a unique opportunity to provide support to families through medical and behavioral health care (Krans et al., 2015; Mascola et al., 2017). Indeed, many women are motivated to seek substance use treatment during pregnancy (Asta et al., 2021) and enter and maintain recovery postpartum (Frazer et al., 2019; Goodman et al., 2020).

In response to this opportunity for treatment engagement, there has been a call for a comprehensive and compassionate response to address contextual factors contributing to OUD and support families impacted by the ongoing opioid crisis (Hand et al., 2021). A comprehensive approach to prenatal opioid use (2021) emphasizes the role of poverty, adverse childhood experiences, historical trauma, and stigma on the understanding of the context of perinatal OUD. The model describes a treatment model which includes: medication for OUD, behavioral health care, patient navigation prenatal/well-child care, psychiatric care, education and employment, parenting development, and other services.

Though this paper will emphasize the role of biobehavioral mechanisms in opioid use, it is essential to consider this in the context of a comprehensive treatment approach and to knowledge the role of poverty, trauma, and stigma in perinatal opioid use and parenting behavior. In particular, pregnant women with OUD have high rates of adverse childhood experiences. One study conducted by Gannon et al. (2021) found treatment-seeking pregnant women with OUD (*N*=152) self-reported an average of 4.3 adverse childhood experiences (SD 2.3; range 0–8). These high rates of adverse childhood experiences require compassionate, trauma-informed care. Black and Hispanic women with OUD may encounter even more barriers to completing substance use treatment compared to White counterparts (Suntai, 2021) due to the intersections of racism and stigma. Stigma in many healthcare, criminal justice, and child welfare systems (Stone, 2015) has led to punitive policies and practices towards pregnant women with OUD which can deter women from seeking and continuing with treatment September 5, 2021 10:19:00PM. Though outside of the scope of this article, the impacts of poverty, trauma, and stigma are essential to understanding the experiences of pregnant women with OUD and any proposed intervention and we ask readers to keep this context in mind.

The current opioid epidemic presents a dire need for effective interventions to address perinatal opioid use and promote wellbeing among opioid-using mothers and their infants. There are several existing interventions demonstrating promise in promoting attachment and positive parenting behaviors in substance-exposed mother–infant dyads, including Attachment and Biobehavioral Catchup Project (Berlin et al., 2014), Mom Power (Muzik et al., 2015, 2016), the Mothers and Toddlers Program (Suchman et al., 2012), and Mothering from the Inside Out (Suchman, 2016) and Mindfulness-Based Parenting (MBP; see below; Duncan et al., 2009). Though a comparison of these interventions is outside the scope of this article, we would like to identify one limitation of these approaches. Specifically, they target women and children in the postpartum period. Focusing on attachment and parenting behavior during pregnancy may capitalize on enhanced motivation for change in the prenatal period. In this manuscript, we address this limitation, present a conceptual model of the mechanisms underlying perinatal opioid use and child well-being, and consider how the use of a MBI, Mindfulness-Oriented Recovery Enhancement (MORE), may address the mechanisms outlined in the proposed model during pregnancy.

## A Dyadic Model Linking Maternal Stress, Coping, and Opioid Use to Parenting Behavior and Child Well-Being

**Figure 1:**
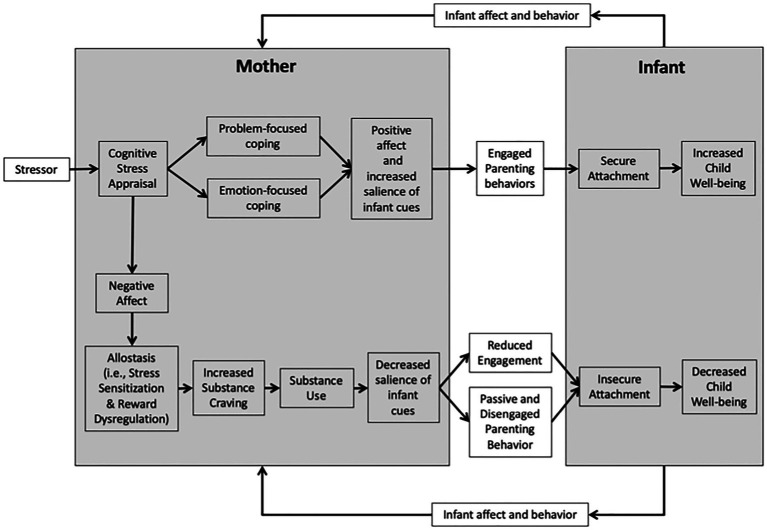
Mechanisms of maternal substance use and child well-being model. This process model represents the connections between perinatal opioid use, parenting behaviors, and infant attachment development. As a mother encounters a stressor, her cognitive appraisal of the situation can drive adaptive coping, leading to positive affect, and engaged parenting behaviors. Alternatively, a negative cognitive appraisal may result in negative affect, promoting the process of allostasis—resulting in increased sensitivity to stress and dysregulation of reward processes. Allostatic load may lead to increased substance craving and substance use, which, over time, results in a decreased salience of infant cues, reduced engagement with the infant, and passive and disengaged parenting behavior. Gradually, the infant may develop an insecure attachment, being unable to rely on his mother’s response. Insecure attachment may then lead to an overall decrease in the child’s well-being. This model also highlights the process of dyadic synchrony. The infant’s affect and behavior also influence the mother’s affect and behavior. As the child learns that the mother’s behavior is unreliable, she may reach out to her mother through behavior that can be interpreted as stressful by her mother, which may reinforce her mother’s cycle of substance craving and use.

Neurobiological and psychological models shed light on the mechanisms underlying the development and maintenance of OUD and interactions between opioid use and parenting. Here we provide an integrated model (depicted in Figure 1) of perinatal opioid use, parenting behavior, infant attachment, and child well-being that unites Lazarus and Folkman’s transactional model of stress and coping (Lazarus and Folkman, 1984), Garland, Boettiger, and Howard’s model of the risk chain linking stress to addictive behavior (Garland et al., 2011), Koob and Volkow’s model of the neurocircuitry of addiction (Koob and Volkow, 2016), Rutherford and Mayes’ reward-stress dysregulation model of addicted parenting (Rutherford and Mayes, 2017), and Bowlby’s attachment theory (Bowlby, 1988). For this paper, we define child well-being in relation to five distinct domains: physical, psychological, cognitive, social, and economic (Pollard and Lee, 2003).

Our model highlights biobehavioral processes linking negative stress appraisals to parenting behavior and child well-being. Negative stress appraisals may lead to negative affect, stress sensitization, reward dysregulation, and ultimately opioid craving and use. Over time, recurrent opioid use may lead to reduced engagement and passive/disengaged parenting behavior, and subsequently, insecure attachment and decreased child well-being. Altogether, opioid use may set off a reciprocal relationship wherein some mothers and infants negatively impact one another. Proposed mechanisms to target with intervention may include negative stress appraisals, stress sensitization, reward dysregulation, substance craving, and parenting behavior. The ultimate goal of developing this model is to inform the development and adaptation of behavioral interventions for opioid-exposed mother–infant dyads.

### Stress and Cognitive Appraisal

The initial stages of our model are informed by Lazarus and Folkman’s (1984) transactional model of stress and coping. Lazarus and Folkman define stress as “a particular relationship between the person and the environment that is appraised by the person as taxing or exceeding his or her resources and endangering his or her well-being” (Lazarus and Folkman, 1984, p. 19). This process of appraisal refers to the evaluation of meaning or significance and is shaped by individual commitments (e.g., what is important to individuals, what has meaning for them, and what is at stake). These commitments motivate individual choices and behavior in an attempt to maintain or achieve goals. When an individual appraises an encounter with a stressor as exceeding their resources, the stress reaction results. Stress can be defined as any situation that requires an individual to respond and adapt. An individual’s response to stress is determined by their appraisal of the situation. If a person appraises the circumstances as harmful and overwhelming their capacity to cope, this can result in distress (i.e., “bad stress”). Alternatively, if the person appraises the circumstances as manageable, this can result in eustress (i.e., “good stress”), which can lead to increased resilience, or the ability to weather hardships. It is important to note here that mothers with OUD report higher perceived parenting stress compared to mothers without OUD (Bagner et al., 2009).

### Problem-Focused and Emotion-Focused Coping

In response to appraising a situation as stressful, a person may then engage in coping—that is, cognitive and/or behavioral efforts to manage or resolve the stressor. Coping may be problem-focused or emotion-focused (Lazarus and Folkman, 1984). Problem-focused coping involves problem-solving and drawing on resources (e.g., social, financial) to resolve the problem or improve the situation. Alternatively, or in addition to problem-focused coping, the person may utilize emotion-focused coping to address emotional distress. One emotion-focused coping skill is *reappraisal*—reinterpreting the meaning of the stressor to reduce emotional distress. We will discuss reappraisal at greater length below. Successful problem-focused or emotion-focused coping may lead to positive affect (Aldwin and Revenson, 1987). When coping fails, an individual may experience increased negative affect, which fuels allostasis, the biological process which occurs as the body responds to stress to regain homeostasis, leading to allostatic load (see below; Sterling and Eyer, 1988).

### Allostatic Load Leads to Stress Sensitization, Reward Dysregulation, and Craving

As a result of a negative stress appraisal, the sympathetic nervous system (SNS; i.e., the branch of the autonomic nervous system that coordinates the “fight/flight” response) is engaged. When an individual negatively appraises a situation, leading to emotional distress, the adrenal glands release epinephrine and norepinephrine, activating the sympathetic-adrenal-medullary and the hypothalamic-pituitary-adrenal (HPA) axes, and increasing heart rate and respiration. This physiological stress response can be adaptive in the short term, motivating a person to engage in activity leading to homeostasis, or a more stable equilibrium. However, over time, chronic stress can result in allostatic load (i.e., “wear and tear” on the body) that can then lead to increased vulnerability to disease and developmental disorders (McEwen and Wingfield, 2003; McEwen and Gianaros, 2011). How individuals appraise these stressors can lead to adaptive coping, which can reduce stress-induced negative affect and improve SNS recovery (Larsen and Christenfeld, 2010). However, an individual’s ability to regulate their emotions through adaptive coping is influenced by the neurobiological effects of recurrent opioid use.

Recurrent opioid use produces neurobiological effects on reward and stress circuitry in the brain (Volkow et al., 2016; Koob, 2019). Koob and Volkow’s (2010, 2016) model of the neurocircuitry of addiction divides the process of drug addiction into three recurring stages: (1) *binge/intoxication*, (2) *withdrawal/negative affect*, and (3) *preoccupation/anticipation*. The binge/intoxication stage is accompanied by neuroadaptations in the basal ganglia that impact the perceived salience of drug-related versus natural rewards. These neuroadaptations are a result of intoxication caused by an increase in several neurotransmitters and neuromodulators (Koob and Volkow, 2016), including dopamine in the ventral striatum and ventral tegmental area, which produce euphoric effects and positively reinforce drug use (Caine et al., 2007). Over time and with recurrent use, mesocorticolimbic brain systems become sensitized to drug cues (e.g., a needle, a pill bottle, a specific place; Schultz et al., 1997; Robinson and Berridge, 2008; Berridge, 2012), and these cues (as opposed to the drug itself) begin to activate *wanting* without *liking*, which propels compulsive behavior (Robinson and Berridge, 2001). Drug cues are thought to activate drug use action schemas, or conditioned responses that initiate a series of unconscious, automatic drug use behaviors (Tiffany, 1990). Over time, drug use action schemas and automatic, or habitual, drug use behavior lead to attentional bias toward drug cues, during which attentional resources are drawn toward drug-related cues (Field and Cox, 2008). Individuals then develop tolerance and begin to increase use, which leads to the second stage of the development of addiction.

The second stage of Koob and Volkow’s (2010, 2016) model, withdrawal/negative affect, is associated with changes in the extended amygdala and negative emotional states, including increased sensitivity to stress and loss of interest in natural rewards. When not using the drug, individuals experience intense emotional and physical discomfort, or symptoms of withdrawal. As addiction dysregulates the neural circuitry implicated in reward processing, it decreases the salience or perceived value of nondrug rewards (Garavan et al., 2000; Volkow et al., 2010). During the perinatal period, these neurobiological changes may interfere with the neurocircuitry responsible for maternal bonding behaviors (Wallin et al., 2021). Prolonged drug use also affects stress circuitry, activating the HPA axis to release molecular mediators of antireward (Koob and Le Moal, 2008), including cortisol, corticotropin-releasing factor, and adrenocorticotropin hormone, producing aversive states during withdrawal or extended abstinence. This overproduction of the stress response systems leads to the final stage of Koob and Volkow’s (2010, 2016) model—craving, or a preoccupation with obtaining and using opioids as a means of allaying the resultant dysphoria.

The third and final stage of Koob and Volkow’s (2010, 2016) model, preoccupation/anticipation (i.e., craving), is a result of changes in the prefrontal cortex that lead to deficits in executive functioning. Tiffany’s model suggests that substance craving is triggered when a drug use action schema is initiated and the person is hindered from engaging the automatic drug use behavior. When craving is triggered by a drug cue and a drug use action schema is triggered, the prefrontal cortex is activated (Lee et al., 2005; Risinger et al., 2005; Volkow et al., 2005; Jasinska et al., 2014; Kober et al., 2016). Craving, stress sensitization, and reward dysregulation can lead to increased opioid use, which fuels this cycle. Reward dysregulation, in particular, may affect the perceived salience of infant cues and, in turn, influence parenting behavior (Brancato and Cannizzaro, 2018; Cataldo et al., 2019; Swain and Ho, 2019).

### Salience of Infant Cues and Parenting Behavior

Decades of animal and human research on parenting has revealed an “intricate interplay of numerous neural, mental, and behavioral processes of perception, motivation, affect, cognition, … and motor performance in shaping the mother’s behavior to engage in a selective and enduring reciprocal emotional relationship with [her child]” (Pereira and Ferreira, 2016, p.72). This dynamic, interactive process occurs within the dyad, with reciprocal influences between infants and mothers. Sensitive mothering requires mothers to attend to, interpret, and respond appropriately to infant cues. Although mothering is impacted by genetics, mothers’ childhood experiences, and culture (Keller et al., 2008), here we will focus primarily on the motivational, affective, and cognitive processes that affect the mother–infant relationship.

Coordination between motivational, affective, and cognitive processes rely on hormones (Bridges, 2015). Changes in hormones during pregnancy and after birth affect brain functioning (Featherstone et al., 2000; Keyser-Marcus et al., 2001; Shingo et al., 2003; Rasia-Filho et al., 2004; Leuner and Gould, 2010; Larsen and Grattan, 2012) and are responsible for early bonding between mothers and infants (see Bridges, 2015). Specifically, oxytocin and prolactin are thought to increase the salience of infant cues (i.e., their value or motivating quality), increase reward experience from interacting with the infant, and induce positive affect (Numan et al., 2006). Higher oxytocin levels have been found to be associated with mothering behaviors such as shared gaze, vocalizations, positive affect, and affectionate touch (Feldman et al., 2007, 2010). Oxytocin levels were also found to correlate with activation in these parts of the brain when mothers were exposed to photos of their babies (Feldman, 2015). Findings from experimental studies are mixed. In one study, when depressed postpartum mothers were given internasal oxytocin, it did not increase the sensitivity of their interactions with their babies, but it did improve their protective behavior (Mah et al., 2017). In a study of brain activation, oxytocin administration was found to be associated with reduced activation in stress systems of the brain (i.e., amygdala, insula, and inferior frontal gyrus) when exposed to infant crying (Riem et al., 2011). Oxytocin administration was also found to be associated with increased salience of infant laughter (Riem et al., 2012).

In addition to oxytocin, cortisol plays a role in mothering behavior. During the first week after birth, hormones (specifically cortisol) originating in the HPA axis also appear to be associated with maternal responsiveness. Corter and Fleming (1990) hypothesize that in this first week, activation of the HPA axis may enrich the perception of salience of infant cues and, as a result, attention to cues. Though, continued heightened activity in the HPA axis later in the postpartum period may be inversely associated with mothering behaviors (Krpan et al., 2005). Oxytocin and cortisol impact the activation of brain networks implicated in mothering behavior.

Human and animal studies have revealed that mothering is influenced by the coordination of multiple brain structures known as the Maternal Brain Neurocircuit (MBN; see Swain and Ho, 2019 for an extensive review of the model). The MBN includes orbitofrontal and prefrontal cortices, bed nucleus of the stria terminalis, amygdala, hippocampus, medial preoptic area, nucleus accumbens, and ventral tegmental area (Kendrick et al., 1997; Numan et al., 2006; Atzil et al., 2011; Barrett and Fleming, 2011; Olazábal et al., 2013; Moses-Lolko et al., 2014). This neural network is thought to subserve a mother’s capacity to perceive and respond to her infant’s needs by coordinating multiple neurocognitive processes, including attention, memory, empathy, decision-making, and stress reactivity (Pereira and Ferreira, 2016). The medial preoptic area, in particular, is critical to effective parenting, as it integrates and coordinates the mothering behavior according to the age of the child (Numan, 1974; Jacobson et al., 1980; Gray and Brooks, 1984; Cohn and Gerall, 1989; Lee et al., 2000; Arrati et al., 2006; Perrin et al., 2007; Pereira and Morrell, 2009). In the context of parenting, the medial preoptic area communicates with nucleus accumbens to regulate motivation; amygdala, bed nucleus of the stria terminalis, and medial prefrontal cortex to regulate affect and cognitive functions (e.g., attention, behavioral flexibility, and working memory; Pereira and Ferreira, 2016). These same neurocognitive processes are also impacted by recurrent opioid use.

Swain et al. (2014) suggest that to the extent that opioid use and mental health disorders dysregulate reward circuitry in the brains of mothers, this process may impact the salience of infant-related rewards, leading to challenges in emotion regulation, maternal responsiveness, attachment, and infant development. In support of this hypothesis, a recent pilot functional magnetic resonance imaging (fMRI) study of resting state function in mothers receiving buprenorphine for OUD (*n*=32) compared to mothers without OUD (*n*=25) found an association between problems with maternal bonding (measured by the Postpartum Bonding Questionnaire) and connectivity in the MBN (Swain and Ho, 2019). They also found buprenorphine treatment may mitigate this risk of bonding deficits.

Rutherford and Mayes (2017) have outlined a model explaining the overlapping brain circuitry involved in the neurobiology of parenting behavior and the etiology of addiction. Research indicates that viewing infant faces is rewarding for both parents and nonparents, activating the nucleus accumbens (Glocker et al., 2009) and other reward circuits in the brain (Rutherford et al., 2011, 2013), seemingly as a way to attract potential caregivers (Kringelbach et al., 2016). They identify brain regions associated with reward (i.e., prefrontal cortex, ventral tegmental area, and nucleus accumbens) and stress (i.e., HPA axis and extended amygdala) that are implicated in the development of addiction, as well as the perception of infant cues and interaction with infants (Rutherford et al., 2011). Their model posits that as substance use dysregulates reward circuitry in the brain of a mother, she experiences a decrease in the salience of infant-related rewards (i.e., she becomes less responsive and less motivated by her infant’s crying and/or smiling).

Findings from three fMRI studies support this hypothesis. Landi et al. (2011) found that mothers using substances demonstrated less brain activation in the prefrontal and limbic regions of the brain compared to mothers not using substances when presented with images of unfamiliar infant faces. Kim et al. (2017) utilized a similar paradigm, but with images of women’s own babies along with photos of unknown infants. They found reduced reward responses in hypothalamus, ventral striatum, and ventromedial prefrontal cortex among women enrolled in inpatient substance use programs compared to women without SUD. Finally, Rutherford et al. (2020) utilized a similar paradigm as Kim et al. (2017)—and found that compared to non-substance-using mothers, mothers using substances demonstrated greater activation in superior medial frontal, inferior parietal, and middle temporal regions when viewing their own infants’ faces rather than unknown infant faces. As such, for substance-using parents, caregiving is less rewarding and more stressful (Rutherford et al., 2011, 2013). We have integrated the reward-stress dysregulation model into our model and expanded on these ideas to consider how parenting behaviors impact attachment.

### Attachment and Child Well-Being

A mother’s ability to attune to her infant, regulate her own physical and emotional experience, and respond appropriately to her infant influences the quality of an infant’s attachment (Bowlby, 1988; Sroufe, 1988). Bowlby’s (1988) attachment theory states that humans are wired to connect within intimate relationships and that infant relationships with caregivers are particularly influential on development and future relationships. There are four types of attachment: secure, avoidant, resistant, and disorganized. Secure attachment develops as a result of consistent, sensitive caregiving, (i.e., providing physical care, emotional communication, and affection in response to infant cues; Ainsworth et al., 1978). In response to attentive, engaged caregiving, infants learn to trust their caregivers, operationalized as children seeking proximity to attachment figures when they experience distress (Ainsworth et al., 1978). Parenting quality can lead to epigenetic changes that influence the brain systems underlying children’s ability to regulate stress and emotion (McGowan et al., 2009). Since infants are unable to self-regulate, a secure attachment with a trusted caregiver can result in co-regulatory stress regulation, buffering the infant’s HPA axis in response to stressors (Schore, 2005). One method of measuring HPA axis activation is salivary cortisol. Nachmias et al. (1996) found elevations in cortisol among insecurely, but not securely attached toddlers when both groups were exposed to a series of stressors. Ahnert et al. (2004) also found significantly greater increases in cortisol responses of insecurely-attached toddlers compared to securely attached toddlers when visiting a new child care center. As children develop, they use attachment figures as a “secure base” from which they can explore the world and take risks. Children with secure attachments are more resilient and self-reliant than their insecurely attached peers (Sroufe, 2005). Avoidant attachment and altered HPA axis function have been found to be associated with impaired social, psychological, and neurobiological functioning (i.e., behavior problems; Snoek et al., 2004; Van Bokhoven et al., 2005a,b; Fearon and Belsky, 2011), anxiety, depression, and post-traumatic stress disorder (Heim et al., 1997; Nemeroff, 2004; Gunnar and Quevedo, 2008), as well as negative outcomes related to childhood school achievement and peer social status (Schore, 1994, 2001).

These findings have implications for perinatal opioid use. As discussed in the introduction, studies have found an association between adverse childhood experiences and illicit substance use in pregnancy (Chung et al., 2010; Leeners et al., 2014; Currie and Tough, 2021; Racine et al., 2021). Given the increased likelihood of experiencing abuse and neglect in childhood, mothers who struggle with OUD may be more likely to exhibit insecure attachment relationships, but research on this topic is limited.

### Infant Affect and Behavior

In addition to considering the impact of parenting behaviors on attachment, we have modeled the reciprocal relationship between infants and mothers. An infant’s symptoms of opioid withdrawal (e.g., inconsolable crying) can be particularly challenging for caregivers, requiring caregivers to regulate negative emotions stemming from caring for the infant in distress. Emotion regulation has been defined as the capacity to control the experience or expression of positive and negative emotions (Gross, 1998, 2015). Children learn emotion regulation strategies from interacting with their caregivers (Fox, 1998), and children’s behavior and affect similarly affect their caregivers’ responses. This dynamic dyadic system is known as emotion co-regulation and is operationalized as the dyad’s shared gaze, complementary affective states, verbal turn-taking, and interactive behavior (Tronick et al., 1977; Dumas et al., 1995; Cole et al., 2003; Deater-Deckard et al., 2004; Lavelli and Fogel, 2005). Co-regulation (e.g., a mother vocally soothing her infant) is a component of sensitive caregiving that promotes resilience among children (e.g., Gerwitz et al., 2008). Dyadic synchrony can help a child learn to self-regulate through the mechanisms described above (Harrist and Waugh, 2002).

Behavioral interventions for perinatal opioid use should address the aforementioned mechanisms that connect opioid use to child well-being. As described above, MBIs are demonstrating promise in addressing these mechanisms. Below, we present evidence supporting the use of one MBI, MORE, with women who are pregnant and have been diagnosed with OUD.

## MBIS Address Substance Use and Promote Attentive Caregiving

Mindfulness is conceptualized as a *practice*, a *state*, and a *trait*. The two primary practices of mindfulness are focused attention and open monitoring (Lutz et al., 2008). Focused attention practice involves a repeated process of first sustaining attention on an object, then acknowledging distractions, and finally, redirecting attention to the object. During open monitoring practice, an individual attends to passing thoughts, emotions, and physical sensations as well as the field of awareness in which mental contents occur. These practices can induce the state of mindfulness (e.g., Lau et al., 2006), a state of awareness during which one cultivates an attitude of acceptance, openness, curiosity, and detachment. Overtime, invoking the state of mindfulness through mindfulness practice leads to the development of the trait of mindfulness (Kiken et al., 2016), or the propensity to act mindfully in everyday life (e.g., Baer et al., 2006). Increases in trait mindfulness as a result of participating in MBIs have been found to be associated with psychological health benefits (Carmody and Baer, 2008; Shapiro et al., 2008; Shahar et al., 2010).

MBIs are demonstrating promise in treating SUDs broadly and OUD specifically. Interventions include Mindfulness-Based Relapse Prevention and MORE. Randomized controlled trials (RCTs) support the use of MBIs to treat alcohol use disorder (Garland et al., 2010; Kamboj et al., 2017; Cavicchioli et al., 2018), stimulant use disorder (Glasner-Edwards et al., 2017), and opioid misuse/OUD (Garland et al., 2014c). A (Li et al., 2017) meta-analysis of RCTs of MBIs for substance use found mindfulness treatment significantly reduced substance use post-treatment [−0.33, 95% CI (−0.88, −0.14)] compared to control conditions.

There is a growing body of evidence supporting the role of mindfulness practices in reducing anxiety, depression, and stress during pregnancy (Duncan et al., 2009; Dhillon et al., 2017; Babbar et al., 2021). This research is expanding to focus on the unique context of perinatal substance use. A research team based in the Division of Maternal Addiction Treatment Education and Research are leading in the field of MBIs for mothers with OUD. In an observational study (*N*=160), Gannon et al. (2017) evaluated the impact of a trauma-informed MBP intervention on parenting quality as measured by the Keys to Interactive Parenting Scale (Comfort and Gordon, 2006). The MBP intervention is based in the model of mindful parenting (Duncan et al., 2009), which emphasizes the role of attention, nonjudgment, compassion, self-regulation, and awareness. The team found that the MBP intervention led to clinically significant improvements in the quality of parenting behaviors. The team also found that the MBP intervention resulted in a significant decrease in general stress (as measured by the Perceived Stress Scale-10; Cohen et al., 1983), parental distress [as measured by the parenting stress index-short form (Abiden, 1995; Short et al., 2017)], and depression symptoms (Alexander et al., 2019).

The model of mindful parenting on which the MBP intervention is based (Duncan et al., 2009) hypothesizes how mindfulness may promote adaptive parenting behaviors. In early infancy, caregivers must be attentive to cries and other behavioral signs of distress or discomfort. This is particularly important for mothers of infants experiencing withdrawal symptoms who require more extensive care. The second dimension of mindful parenting, nonjudgmental acceptance of self and child, is relevant to mothers who are prescribed medication for OUD (e.g., buprenorphine) or used opioids while pregnant due to frequent reports of shame and self-judgment regarding substance use (Covington, 2008). Emotional awareness of self and child involves being able to identify emotions within self and child and regulating strong negative emotions. Finally, compassion has been described as the “desire to alleviate suffering” (Lazarus and Lazarus, 1994). Through heightened compassion, parents who practice mindfulness may be more responsive to their infants’ needs. We will now describe the components of one MBI, MORE, and how MORE may address the mechanisms of perinatal opioid use.

## More and the MMT

Mindfulness-Oriented Recovery Enhancement is a sequenced treatment made up of three primary components—mindfulness, reappraisal, and savoring. MORE is based on the *mindfulness-to-meaning theory* (MMT), which provides a dynamic causal model of the mechanisms by which mindfulness promotes positive emotions and the sense of meaning in life (Garland et al., 2015). This promotion of positive emotions is relevant to pregnancy, which for many, can be a time of reprioritizing values and meaning-making (Prinds and Hvidt, 2014). Garland et al. (2015) argue that mindfulness research has been myopically focused on the study of attention regulation as a means of eliminating maladaptive behaviors, emotions, and cognitions, while neglecting the historical purpose of these practices—namely, fostering eudemonic states through positive emotion regulation and the development of prosocial behavior. The MMT posits that mindfulness practice can promote metacognition, altering the quality of awareness and thereby enabling positive reappraisal, positive affect, and adaptive behavior (Garland et al., 2015).

In MORE, participants engage in mindfulness practices like the body scan, mindful breathing, and open-monitoring to strengthen executive functioning and attentional networks in the brain. Participants then apply this enhanced cognitive control capacity to the process of reappraisal, a form of emotion-focused coping utilized to restructure maladaptive cognitions and decrease stress. Finally, participants utilize these mindfulness skills to practice savoring, the intentional process of focusing on and enhancing responses to naturally rewarding experiences in life (Bryant and Veroff, 2007; Garland, 2016).

**Figure 2:**
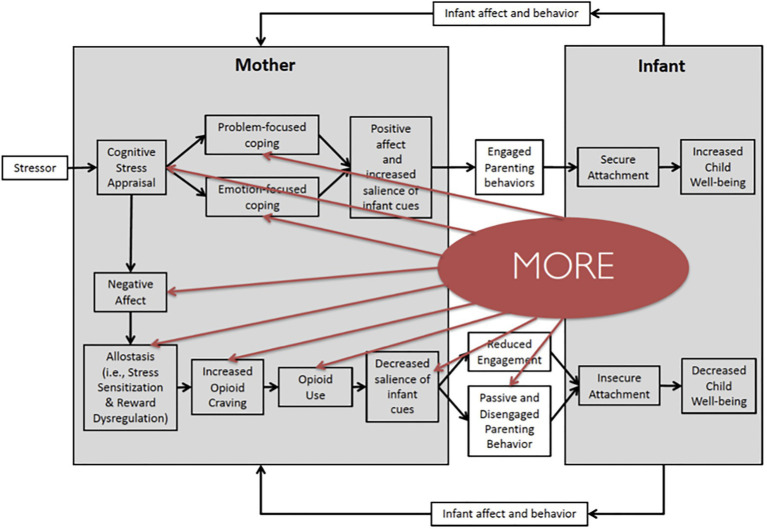
Mindfulness-Oriented Recovery Enhancement (MORE) targets mechanisms of maternal substance use and child well-being. MORE may interrupt several key processes in the proposed model of perinatal opioid use and child well-being. MORE promotes the process of *mindful reappraisal*, whereby a mother can change her perspective on a stressor to decreased her negative affect. MORE also fosters problem-focused and emotion-focused coping, during which a mother uses mindfulness practices to cope with emotional distress. MORE also targets the process of allostasis, reduces sensitization to stress, and increases sensitization to reward. MORE also provides strategies to cope with substance craving, decreasing the likelihood of substance use. Finally, *mindful savoring* may help women increase the salience of infant cues and promote engaged parenting behavior.

Mindfulness-Oriented Recovery Enhancement has demonstrated efficacy across multiple RCTs of chronic opioid users and opioid misusers, as well as patients with OUD. In the first Stage 2 RCT of MORE (*N*=115) for opioid misuse (Garland et al., 2014c), MORE significantly decreased opioid misuse behaviors indicative of OUD (↓ occurrence of OUD by 63%) relative to a supportive group psychotherapy (SG) control condition. A second Stage 2 RCT (*N*=95; Garland et al., 2019c) replicated these results, demonstrating again that MORE opioid misuse (*p*=0.027, *d*=0.64), and opioid use (*p*=0.006, *d*=1.07; Garland et al., 2020). In a third Stage 1 RCT of people with OUD (*N*=30), combining MORE with methadone maintenance therapy decreased days of heroin and other drug use (*F*=4.72, *p*=0.04) to a greater extent than methadone plus usual care (Cooperman et al., 2021). Finally, in a new, full-scale RCT (*N*=250), MORE reduced opioid misuse by 46% at the 9-month follow-up. Taken together, findings from these trials (total *N*=490) demonstrate MORE’s efficacy for decreasing addictive use of opioids. In light of MORE’s efficacy, below, we expand on these mechanisms by which MORE may address perinatal OUD and review the supporting evidence (see Figure 2).

### MORE Addresses Attentional Bias, Cue-Reactivity, and Craving

Evidence from mechanistic studies and RCTs support the hypothesis that MORE reduces attentional, physiological, and subjective reactivity (i.e., craving) to drug cues. In a pilot RCT (*n*=53) with alcohol-dependent adults in an inpatient setting, MORE significantly reduced attentional bias to alcohol-related cues during a dot probe task compared to a support group (SG) control condition (Garland et al., 2010). In the same group, less alcohol attentional bias predicted a lower rate of return to alcohol use (Garland et al., 2012). MORE may decrease attentional bias to drug cues by promoting attention regulation and disengagement from stimuli (Garland et al., 2017a). In support of this theory, MORE was found to significantly reduce opioid attentional bias in a sample (*n*=115) of opioid-treated chronic pain patients compared to a SG (Garland et al., 2017a); and MORE was shown to be associated with reduced opioid cue-reactivity as evidenced by decreased salivation during *in vivo* opioid cue-exposure (Hanley and Garland, 2020).

With regard to subjective craving, mechanistic studies have found that MORE decreases cue-elicited craving (Garland et al., 2014a; Hanley and Garland, 2020). In a study of opioid-misusing chronic pain patients (*n*=115), MORE was found to significantly decrease opioid craving (*p*=0.027; Garland et al., 2014c). Similarly, a pragmatic RCT of men diagnosed with co-occurring disorders (*n*=180) found that MORE led to significantly greater decreases in craving than cognitive-behavioral therapy or usual care (Garland et al., 2016). Finally, analysis of a stage 1 RCT of MORE with methadone-maintained individuals (*N*=30) revealed that MORE significantly reduced craving (*p*<0.001) compared to methadone maintenance as usual control condition (Garland et al., 2019b).

Garland et al. (2014b) argue that mindfulness enhances functional connectivity between prefrontal-parietal metacognitive attentional control networks and subcortical limbic-striatal circuits involved in emotional habits and responses. For pregnant women with OUD, mindfulness practice may decrease attentional bias to opioid-related cues, decreasing cue-reactivity and craving, and thereby allowing for disengagement from automatic, habitual opioid use.

### MORE Enhances Stress Recovery and Reduces Stress Reactivity

In addition to reducing substance attentional bias, MORE may capitalize on increased cognitive control to reduce stress through mindful reappraisal and parasympathetic activation, circumventing stress-induced relapse. The *mindful reappraisal hypothesis* (Garland and Fredrickson, 2019) states that as individuals learn to regulate attention through mindfulness practice, they increase their ability to widen their attention in order to disengage from momentary thoughts, feelings, and sensations and become aware of contextual information that they previously ignored. By widening attention from solely being focused stressful thoughts, feelings, and sensations, individuals can identify more helpful ways of thinking, which then decreases negative emotional reactions. The mindful reappraisal hypothesis as it relates to MORE is supported through findings from mechanistic studies and RCTs. One study found that compared to a SG, participation in MORE was associated with decreases in perceived stress (as measured by the Perceived Stress Scale; Cohen et al., 1983) and increased parasympathetic recovery when exposed to stress-primed alcohol-related stimuli (Garland et al., 2010). In a study of opioid-misusing chronic pain patients (*n*=115), MORE was found to significantly increase reappraisal while decreasing stress arousal (as measured by the Calgary Symptoms of Stress Inventory; Carlson and Thomas, 2007; *p*=0.034) compared to a SG (Garland et al., 2014c). A recent study of MORE compared to a SG found MORE significantly reduced stress (measured by ecological momentary assessment, 0–10 numeric scale in response to the question “How stressed are you right now?”) among a sample (*N*=30) of individuals receiving methadone maintenance treatment compared to a TAU control condition (Garland et al., 2019b). As described above, stress plays a central role in perinatal opioid use, which can prime a return to use and inhibiting attentive mothering behavior. Given the significance of stress, interventions like MORE may improve opioid use and parenting behavior.

### MORE Restructures Reward

The *mindful savoring hypothesis* (Garland and Fredrickson, 2019) posits that mindfulness practice may boost positive emotions and amplify natural reward processing in the brain by facilitating savoring – the process of attending to the pleasurable features of a positive object or event while appreciating one’s own emotional and somatic response to the pleasant event. For example, a parent may savor the experience of rocking their child to sleep The parent may focus on the physical sensation of pressure and warmth, the sound of their child’s breathing, the look of relaxation on their child’s face, or the smell of their child’s hair. They may then focus on the emotions that arise—feelings of calm, closeness, and love for their child. Finally, they would focus on these feelings with the intention of amplifying and absorbing those feelings. Savoring is the focus of a growing body of evidence supporting the use of MORE as a way to address reward dysregulation caused by recurrent substance use. High-frequency heart rate variability (HRV), the beat-to-beat change in heart rate, represents parasympathetic nervous system regulation of the heart (Berntson et al., 1997) and is often used as a measure of central and autonomic nervous system activity (e.g., prefrontal and cingulate cortices and vagus nerve), which are associated with reward responsiveness, attention, and emotion regulation (Thayer et al., 2009; Thayer and Lane, 2009). Among a sample (*n*=115) of chronic pain patients prescribed opioids, MORE, compared to a SG, was found to increase HRV in response to natural rewards, which mediated reductions in craving (Garland et al., 2014a).

In an EEG study of opioid-treated chronic pain patients (*n*=29), MORE was associated with enhanced late positive potential (LPP) toward photos of natural rewards, which was associated with reductions in opioid craving (Garland et al., 2015). Participation in MORE relative to SG was also found to be associated with a shift in relative HRV response towards drug versus natural rewards, with participants demonstrating a decreased response towards drug rewards and a greater response towards natural rewards in an affective picture viewing task (Garland et al., 2017c). This decrease in HRV responsivity to drug rewards and increase in HRV responsivity to natural rewards predicted decreases in opioid misuse in a 3-month follow-up (Garland et al., 2017c). A recent EEG study (Garland et al., 2019a) of opioid-treated chronic pain patients (*n*=135) found MORE (relative to a SG) decreased neurophysiological reactivity to drug-related cues and increasing responsiveness (i.e., LPP) to natural reward cues. This enhanced regulatory capacity was associated with reductions in craving and opioid misuse. Analysis of ecological momentary assessment found that those who participated in MORE were 2.75 times more likely than those in SG to maintain or increase positive effect in day-to-day life, which predicted reductions in opioid misuse (Garland et al., 2017b). Finally, ecological momentary assessment findings from a recent Stage 1 RCT of MORE with individuals (*N*=30) receiving methadone maintenance treatment indicate that MORE significantly increased positive affect (*p*=0.017) compared to a TAU control condition (Garland et al., 2019b).

Here, we would like to highlight two parenting interventions which are demonstrating promise in addressing dysregulation in the MBN—Attachment and Behavioral Catchup (Berlin et al., 2014) and Mom Power (Muzik et al., 2015, 2016). A recent study found greater enhancement of ERP response (N170 and LPP) to viewing emotional faces of children in participants (mothers referred to child protective services) randomly assigned to ABC (*n*=30) compared to a control group (*n*=21) of child protective service-referred women. These findings support the hypothesis that short-term attachment-based parenting interventions may target dysfunction in the MBN (in particular, processing of emotional faces). A pilot study of Mom Power—an attachment-based parenting intervention targeting maternal empathy, reflective functioning and stress reduction—found the intervention to be feasible and acceptable among a group of women with OUD (*N*=68; Muzik et al., 2015, n.d.) to reduce depression, symptoms of posttraumatic stress disorder, and caregiving helplessness among high-risk mothers. A larger community-based RCT (*N*=122) found improvements in mental health and parenting stress for high-risk mothers after participating in MOM Power, in contrast to negative parenting outcomes (i.e., increase parent–child role-reversal) for the control group (Rosenblum et al., 2017). The effects of MOM Power on MBN have been examined with two fMRI studies utilizing a Child Facing Mirroring Task and fMRI (Ho et al., 2020). The authors found that participation in MOM Power led to decreased parenting stress which may have been mediated by changes in left superior-temporal-gyrus, peraqueductal gray, and left amygdala. Altogether, these studies support the hypothesis that attachment-based parenting interventions can address dysregulation of the MBN.

Mindfulness may be one such intervention for women who are pregnant and using opioids to help them cope with aspects of addiction, manage stress, and facilitate savoring and appreciation of pleasant experiences of connection with their children during pregnancy and early parenthood. For instance, using mindfulness to savor the touch of the hand of one’s infant may produce a sense of abiding pleasure, love, and deep interconnectedness that comes to outweigh the pull of drug-related reward. Mindfulness practice during and after pregnancy may lead to positive mental states, positive behaviors, and ultimately the cultivation of a sense of meaning and purpose in life.

## Discussion

Opioid use among pregnant women is a complex issue that warrants increased attention from the scientific community. There appears to be a spectrum of risk stratified by variables such as polysubstance use, tobacco use, low socioeconomic status, trauma history, and comorbid physical and mental health disorders. More research is needed to learn about perinatal opioid use and develop targeted treatments. Mechanistic research indicates that prolonged nonmedical opioid exposure modulates the neurocognitive and neuroaffective processes underlying addiction and produces adverse neurobiological consequences during pregnancy and after birth. As described in the introduction, a compassionate and comprehensive approach is recommended for all women with prolonged exposure to opioids during pregnancy to reduce the risk of a reoccurrence of use. Overall, there is a need for pregnant women to be included in RCTs of integrated interventions.

Our proposed model builds upon the Rutherford and Mayes’ (2017) *reward-stress dysregulation model of addicted parenting* by including processes that can be targeted for intervention (e.g., cognitive appraisal, problem- and emotion-focused coping, and allostasis). This model outlines the underlying mechanisms leading from negative stress appraisals to maternal substance use and decreased child well-being. Appraisals of stressful or perceived stressful circumstances lead to substance craving and use. Recurrent substance use may lead to increased sensitivity to stress and dysregulation of reward, resulting in decreased perceived salience of infant cues, disengaged parenting behavior, and decreased child well-being. As substance use increases in the United States overall as well as with pregnant women, more research is needed to learn the best ways to support women, as well as their infants, in the context of their environments. Clinical trial MORE is one such MBI that may be utilized to address opioid use and psychological distress among mothers during the perinatal period.

## Author Contributions

SR developed the model and wrote the manuscript with support from EC, MR, and EG. All authors contributed to the article and approved the submitted version.

## Funding

This work was supported by the Mind and Life Francisco J. Varela Research Grant Program (PI:Reese) and grant R01DA042033 from the National Institute on Drug Abuse (PI: Garland).

## Conflict of Interest

EG is the Director of the Center on Mindfulness and Integrative Health Intervention Development. The Center provides Mindfulness-Oriented Recovery Enhancement (MORE), mindfulness-based therapy, and cognitive behavioral therapy in the context of research trails for no cost to research participants; however, EG has received honoraria ad payment for delivering seminars, lectures, and teaching engagements (related to training clinicians in MORE and mindfulness) sponsored by institutions of higher education, government agencies, academic teaching and receives royalties from the sale of books related to MORE.

The remaining authors have no conflicts of interest to declare.

## Publisher’s Note

All claims expressed in this article are solely those of the authors and do not necessarily represent those of their affiliated organizations, or those of the publisher, the editors and the reviewers. Any product that may be evaluated in this article, or claim that may be made by its manufacturer, is not guaranteed or endorsed by the publisher.
